# A genetically encoded ratiometric indicator for tryptophan

**DOI:** 10.1038/s41421-023-00608-1

**Published:** 2023-10-31

**Authors:** Rongkun Tao, Kui Wang, Tian-lun Chen, Xin-xin Zhang, Jian-bin Cao, Wen-quan Zhao, Jiu-lin Du, Yu Mu

**Affiliations:** 1 Institute of Neuroscience, State Key Laboratory of Neuroscience, Center for Excellence in Brain Science and Intelligence Technology, Chinese Academy of Sciences, Shanghai, China; 2 Institute of Genetics and Developmental Biology, Chinese Academy of Sciences, Beijing, China; 3 University of Chinese Academy of Sciences, Beijing, China; 4 School of Life Science and Technology, ShanghaiTech University, Shanghai, China; 5 Department of Anesthesiology, Taizhou Hospital of Zhejiang Province affiliated to Wenzhou Medical University, Wenzhou, Zhejiang China

**Keywords:** Biological techniques, Cell biology

Dear Editor,

L-tryptophan is the rarest essential amino acid found in food and cellular uptake of tryptophan is vital for protein synthesis in vertebrate animals^1^. Tryptophan metabolism is emerging as a critical hub for metabolic regulation of immune responses and neural activities through the kynurenine and serotonin pathways^1,2^. Metabolic reprogramming of tryptophan dynamics is highly associated with inflammation^3^, which plays important roles in neurological and cardiovascular diseases, ageing, and cancers^1,2,4,5^.

Invasive approaches, such as high-performance liquid chromatography (HPLC), and genetically encoded fluorescence resonance energy transfer nanosensors (FLIPWs) have been developed to measure tryptophan concentrations^6,7^. However, these methods are either time-consuming or limited by detection range and photobleaching effect^8^, making them unsuitable for detecting intracellular and extracellular tryptophan dynamics in vivo. As a result, we lack a viable approach for quantitative and systemic measurement of tryptophan metabolism in vivo.

**Fig. 1:**
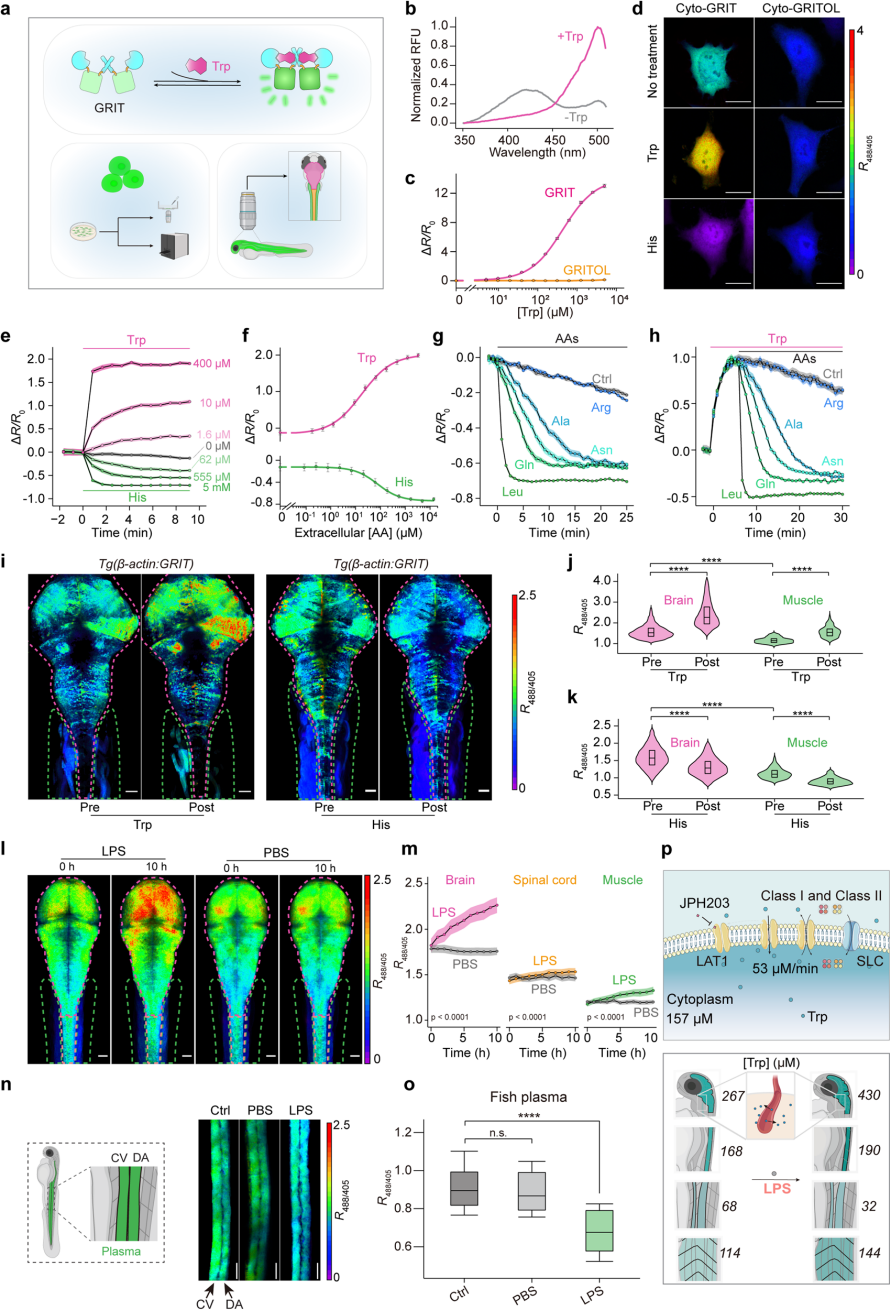
Spatiotemporal visualization of tryptophan in vitro and in vivo with ratiometric GRIT sensor. **a** GRIT sensor allows quantitative measurements of tryptophan in cultured cells and zebrafish. Design and possible working mechanism of GRIT sensor (top). GRIT sensor is composed of TrpR (PDB ID: 1JHG, shown in cyan) and cpSFYFP (PDB ID: 3EVP, shown in green). Tryptophan (Trp, shown in magenta) binding to GRIT sensor elicits a large fluorescence change, enabling quantitative measurements of tryptophan (bottom). **b** Excitation spectra of purified GRIT in the absence or presence of 1 mM Trp, normalized to the major peak intensity at 1 mM Trp. Emission was measured at 530 nm. Two excitation peaks were at 420 nm (Ex1) and 500 nm (Ex2), respectively (indicated by arrows). **c** Excitation ratios of GRIT and GRITOL in the presence of different concentrations of Trp. **d** Fluorescence images of HeLa cells expressing cytosolic GRIT (left) or GRITOL (right) in respond to Trp or His in HBSS buffer. Images are pseudocolored with the ratio of excitation fluorescence at 488 nm and 405 nm (*R*_488/405_). Scale bars, 10 μm. **e** Time course of averaged fluorescence intensity of GRIT in cytosol in response to exogenous Trp or His of absolute concentrations in HBSS buffer. **f** The maximum dynamic ranges were plotted against Trp or His concentrations. **g** Kinetics of GRIT responses in HeLa cells upon addition of 1 mM indicated amino acids. **h** Bidirectional responses of GRIT sensor to the addition of 5 μM Trp and a followed addition of 1 mM indicated amino acids. **i** Representative images of GRIT sensor driven by *beta-actin* in response to 5 mM Trp or 20 mM His. Scale bars, 50 μm. **j**, **k** Group analysis of GRIT responses to Trp (**j**) or His (**k**) (*n* = 4). **l**, **m** Representative images (**l**, maximum projection along the z-axis) and averaged kinetics (**m**) of GRIT signals in brain, spinal cord and muscles of zebrafish treated with LPS or PBS (*n* = 6–8). Scale bars, 50 μm. **n**, **o** Representative fluorescence images (**n**) and mean ratios (**o**) of GRIT protein in plasma of zebrafish caudal vein (CV) and dorsal aorta (DA) with the indicated treatments (*n* = 3–5). Scale bars, 50 μm. **p** The quantification measurements of tryptophan levels and biophysical models of tryptophan in mammalian cells (top) and zebrafish (bottom). Tryptophan enters cells by SLC7A5 (LAT1) and exits from cells in the present of Class I and II amino acids, which could be inhibited by JPH203 (top). During LPS-induced inflammation, plasma tryptophan enters the brain, spinal cord, and muscles (bottom). SLC, solute carrier. Data shown as means ± sem. (n.s. not significant; ****P < 0.0001). *n* = 3–6 independent experiments (**b**–**h**). Two-way ANOVA (**m**) and Student’s unpaired *t-*test for *(***j**, **k**, **o**).

In this study, we fused a circularly permuted superfolder YFP^9,10^ into every insertion site of the flexible loop (site 64–69) of bacterial tryptophan repressor protein TrpR^11^, where has a large conformational change upon tryptophan binding. We then performed linker truncations and screened a biosensor with ~1.5-fold fluorescence responses to tryptophan. Semi-rational design was conducted by focusing on the residues close to the chromophore or linkers, yielding a mutant with ~6-fold response. The GRIT sensor (Genetically encoded Ratiometric Indicator for Tryptophan) was obtained after screening ~8000 mutants in the linker saturated mutagenesis library. We also engineered a GRIT control sensor (GRITOL) without responses to tryptophan (Fig. 1a–c; Supplementary Fig. S1a–e).

GRIT exhibits two excitation peaks near 420 nm and 500 nm and one emission peak near 515 nm. Tryptophan binding elicits a fourfold increase and a 3.3-fold decrease in GRIT fluorescence with excitation at 485 nm and 420 nm, respectively, and a 1300% ratiometric fluorescence change with an apparent tryptophan dissociation constant (*K*_d_) ~ 430 μM (Fig. 1c; Supplementary Fig. S2a–c and Tables S1, S2). GRIT has high selectivity for L-tryptophan and resists to environmental disturbances (Supplementary Fig. S2d–h), such as pH changes. Thus, ratiometric GRIT is a robust tryptophan reporter with high sensitivity and selectivity.

Vertebrate tryptophan transport is mediated by L-type amino acid transporter 1 (SLC7A5, also known as LAT1)^7^. SLC7A5 is modeled as the major exchanger for large neutral amino acids and mediates exchange of tryptophan with other SLC7A5 substrates such as His between cytosol and extracellular milieus. HeLa cells expressing cyto-GRIT sensor showed intense fluorescence, enabling fluorescence measurement by confocal microscopy or microplate reader in HBSS buffer (Fig. 1d; Supplementary Fig. S3a, b). Adding tryptophan increased excitation ratio of cytosolic GRIT by 2-fold, while His addition decreased signal by ~74% (Fig. 1d–f). Cytosolic free tryptophan level was estimated to be 157.2 ± 17.4 μM based on the apparent occupancy^9^, comparable to HPLC/MS measurements (207.3 ± 8.2 μM) (Supplementary Tables S2, S3).

We found that intracellular tryptophan pool is finely regulated by exogenous tryptophan and other specific amino acids, which can be classified into three groups (Fig. 1g, h). Class I amino acids, reported as SLC7A5 substrates^7^, induce similar and rapid tryptophan export kinetics (~37 μM/min). Class II, comprising five uncharged amino acids (Ala, Asn, Gln, Ser, and Thr), export tryptophan at varying rates (~6.8–14 μM/min) but much slower than Class I. Cancer cell metabolism is reprogrammed to be addicted with Gln^5^, thus it would be interesting to investigate how tumors balance tryptophan availability and Gln addiction to support their growth, such as by tryptophan-to-phenylalanine substitutions^12^. Class III amino acids (Arg, Asp, Glu, Gly, Lys, and Pro), being charged or nonpolar, exhibit no significant impact on tryptophan dynamics (Fig. 1g, h; Supplementary Fig. S3c and Table S4). Inhibition of SLC7A5 with a specific antagonist (JPH203) blocks tryptophan transport induced by Class I and Class II amino acids (Supplementary Fig. S3d). These results, along with the mRNA and protein levels of SLC7A5 (Supplementary Fig. S3e, f), indicate that SLC7A5 is the major tryptophan transporter in HeLa cells.

We treated HeLa cells with 10 ng/mL IFN-γ and this stimulation could significantly upregulate expression of indoleamine 2,3-dioxygenase 1 (IDO1) (Supplementary Fig. S3g), consistent with previous reports^13^. We observed a time-dependent decrease in fluorescence ratios of GRIT sensor, indicating a depletion of cytosolic tryptophan pool after 48 h treatment and this process was blocked by 1 μM IDO1 inhibitor Epacadostat (Supplementary Fig. S3h, i). These findings demonstrate GRIT sensor could detect and quantify intracellular tryptophan decay and highlight the role of IDO1 in tryptophan catabolism.

Optical transparency of larval zebrafish allows elucidating systemic tryptophan dynamics by expressing GRIT sensor broadly (Supplementary Fig. S4a and Video S1). To calibrate tryptophan levels in vivo, we measured dynamic range of GRIT sensor in isolated primary zebrafish cells, using methods similar to those applied in HeLa cells (Supplementary Fig. S4b, c and Tables S2, S3).

Bath application of 5 mM tryptophan led to robust increases in excitation ratios of GRIT in single cells and across the whole body within 3 h, but no increase was observed in GRITOL-expressing animals (Supplementary Fig. S4d–h and Video S2). The intracellular free tryptophan levels in intact brain (206.1 ± 21.8 μM) and muscles (111.0 ± 6.5 μM) showed increases of 140% (494 ± 64.1 μM) and 91% (211.7 ± 19.7 μM) following tryptophan addition, respectively. Adding 20 mM His decreased intracellular tryptophan levels to 137.9 ± 13.8 μM in brain and 77.8 ± 7.9 μM in muscles (Fig. 1i–k; Supplementary Fig. S4i–k and Table S3). GRIT sensor revealed a notable difference and bidirectional changes of intracellular tryptophan levels in brain and muscles of larval zebrafish, possibly reflecting varying tryptophan metabolic pathways and functions across tissues.

Inflammation is a common hallmark of many diseases^1,2,4,5^, and is linked to tryptophan metabolism disruptions^3^. To characterize tryptophan dynamics in response to inflammation, we injected lipopolysaccharide (LPS) into the yolk of zebrafish larvae to induce inflammatory responses. This was evidenced by an ~1.6-fold macrophage accumulation at the yolk, and an ~13-fold increase in reactive oxygen species level (Supplementary Fig. S5a–f). We observed a systemic tryptophan redistribution among different tissues during inflammation. Intracellular tryptophan concentrations increased by 60% in brain (267.3 ± 22.1 μM to 430.2 ± 38.5 μM), 26% in muscles (114.5 ± 4.9 μM to 144.3 ± 8.4 μM), and 13% in spinal cord (167.5 ± 8.7 μM to 190.1 ± 9.3 μM) (Fig. 1l, m). Interestingly, plasma tryptophan level decreased by nearly half in LPS-treated zebrafish (68.1 ± 3.8 μM to 31.6 ± 3.0 μM) (Fig. 1n, o; Supplementary Table S5). None of these changes were observed in PBS-injected GRIT-expressing larvae or GRITOL-expressing controls (Supplementary Fig. S5h–l). Inflammation-induced plasma tryptophan entry into tissue cells may be an important way to balance functions of immune systems and neural behaviors under pathological inflammation through kynurenine and serotonin pathways^2,14^.

We provided a comprehensive comparison between GRIT and existing tryptophan measurement methods (Supplementary Table S6). GRIT biosensor with a smaller protein size exhibits over 30-fold higher dynamic range in cells and a reduced photobleaching effect compared to FLIPW^7^. These advantages make GRIT a superior tool that can be applied in various scenarios and long-term recordings. We presented quantitative measurements of tryptophan in mammalian cells and zebrafish (Fig. 1p). We found that plasma tryptophan enters the brain during inflammation and reduced plasma tryptophan levels could serve as a biochemical marker of inflammation in zebrafish, similar with a previous report in glioblastoma^15^. Considering that inflammation is a hallmark of a variety of diseases^1,2,4,5^, understanding systemic redistribution of tryptophan might provide new insights into metabolic regulation mechanisms underlying these diseases. We anticipate that systemic tryptophan metabolism description in zebrafish could inform human metabolic studies and contribute to development of new therapies for inflammation-related diseases. Overall, GRIT sensor enables a quantitative and systemic investigation of tryptophan dynamics across multiple scales and model organisms, furthering our understanding of its functions under both physiological and pathological conditions.

### Supplementary information

Supplementary information

Supplementary Video 1

Supplementary Video 2
