# Author Correction: A genetically encoded ratiometric indicator for tryptophan

**DOI:** 10.1038/s41421-024-00695-8

**Published:** 2024-06-10

**Authors:** Rongkun Tao, Kui Wang, Tian-lun Chen, Xin-xin Zhang, Jian-bin Cao, Wen-quan Zhao, Jiu-lin Du, Yu Mu

**Affiliations:** 1 Institute of Neuroscience, State Key Laboratory of Neuroscience, Center for Excellence in Brain Science and Intelligence Technology, Chinese Academy of Sciences, Shanghai, China; 2 Institute of Genetics and Developmental Biology, Chinese Academy of Sciences, Beijing, China; 3 University of Chinese Academy of Sciences, Beijing, China; 4 School of Life Science and Technology, ShanghaiTech University, Shanghai, China; 5 Department of Anesthesiology, Taizhou Hospital of Zhejiang Province affiliated to Wenzhou Medical University, Wenzhou, Zhejiang China

**Keywords:** Biological techniques, Cell biology

Correction to: *Cell Discovery* (2023) 9:106

10.1038/s41421-023-00608-1 published online 31 October 2023

In the version of this article initially published, the 2023 Youth Innovation Promotion Association CAS funding was omitted from the Acknowledgements. And the funding code 21171090 should be 32171090.

These corrections have been made in the HTML and PDF versions of the article.

