# Supplementary material for: A genetically encoded ratiometric indicator for tryptophan
Source: Cell Discov. 2023 Oct 31;9:106. doi: 10.1038/s41421-023-00608-1 (PMC10618466; doi:10.1038/s41421-023-00608-1)
Supplement: Supplementary file 1 — Supplementary information [file 41421_2023_608_MOESM1_ESM.pdf]

Supplementary Information

Materials and Methods

Reagents and Drugs

PrimeSTAR Max DNA Polymerase was purchased from TaKaRa; Restriction endonucleases and T4 DNA ligase were obtained from Fermentas; Hieff Clone Plus One Step Cloning Kit was from Yeasen. L-tryptophan, D-tryptophan, L-kynurenine, and lipopolysaccharides (LPS) were all obtained from Sigma-Aldrich. Glutamine and pyruvate were provided from Invitrogen. LAT1 antagonist JPH203 was from Selleck. Other reagents, such as the 19 amino acids, the inorganic salts were of analytical grade and obtained from local suppliers.

Development of Tryptophan Sensors

To make a single fluorophore tryptophan sensor, we inserted the bright, stable circularly permuted superfolder YFP (cpSFYFP) into TrpR and performed several cycles of iterative sensor design and optimization **(Supplementary information, Fig. S1a, b**). TrpR was amplified from the *E. coli* MachI genome and subcloned into the pRSETb vector using BamHI and HindIII. cpSFYFP was made by introducing superfolder GFP mutations[1] into cpYFP from the iNap1 indicator[2]. Site 64-69 of TrpR is a flexible loop, which shows a large conformational change in crystal structures upon tryptophan binding and could tolerate cpSFYFP insertion. The pRSETb-TrpR was split at every combination of two sites between G64 and R69 (15 combinations, **Supplementary information, Fig. S1a, c**) by inverted PCR and fused with cpSFYFP by PstI (Linker1, SAG) and Acc65I (Linker2, GGT), followed by transformation into competent JM109(DE3) cells. Expression of these 15 chimeric proteins was induced by adding 1 mM isopropyl-beta-D-thiogalactopyranoside (IPTG) in Luria-Bertani medium at 20 °C for 24 hrs. Harvested cells were lysed by repeated freezing and thawing, and the supernatants were diluted in detection buffer (10 mM HEPES, 100 mM KCl, pH 7.3) for fluorescence detection. We measured the responses of indicators to different doses of tryptophan and two other aromatic amino acids, phenylalanine and tyrosine. All the insertions were named as Probe and number (Pn), e.g., P2 has the cpSFYFP insertion between G64 and M66 of TrpR. Upon tryptophan addition, three probes (P2, P3, and P8) showed obvious excitation fluorescence ratio changes of approximately 100%, -30%, and 60%, respectively (**Supplementary information, Fig. S1c**). Meanwhile, none of them responded to two other aromatic amino acids (Phe and Tyr).

We then performed empirical truncations sequentially on the N-terminal and C-terminal amino acid linkers flanking cpSFYFP to improve the maximum responses[3] (**Supplementary information, Fig. S1a**). In brief, we removed the N-terminal linker SAG and the first two amino acids of cpSFYFP separately and named the resulting constructs Nx (x is the truncated amino acid number). Next, the most responsive mutants were subjected to C-terminal linker truncations. For instance, in the mutant

denoted by P2-N3C1, three amino acids and one amino acid were deleted from the N-terminus and C-terminus of cpSFYFP in P2, respectively. To simplify the notation, this truncation was also termed P2.4 by adding the serial number after probe P2 (**Supplementary information, Fig. S1d**). We identified four mutants, P2.4, P2.5, P3.4, and P3.5, with improved fluorescence responses (~1.5-fold) in the presence of 1 mM tryptophan (**Supplementary information, Fig. S1d**).

Semi-rational design was performed based on the structure of TrpR (PDB ID: 1JHG) and cpsfYFP (PDB ID: 3EVP). Mutations were focused on the sites V126, P224 and R316. Site V126 (206 relative to GFP) is on the β-sheet of cpFP, and mutations were reported to increase the fluorescence responses of other biosensors[4, 5]. Site P224 (58 relative to GFP) is adjacent to the chromophore, and the pyrrolidine ring of proline might affect the conformational change of cpSFYFP. For the saturated mutations of V126 and P224, we replaced the targeted codon with NNS in primers, in which N means A/T/G/C and S means G/C. Two mutants, V126M and P224T, displayed increases of approximately 20% and 30% in the fluorescence response, respectively (**Supplementary information, Fig. S1b, e**). The positively charged R316 (R69 relative to TrpR) might repulse site R63 and restrict the tryptophan response of the biosensor. Thus, changing R316 to Asp or Glu might increase the sensor response by electrostatic interaction with R63 in TrpR. These semi-rational designs led to the mutant GRIT0 (P2.4-V126M/P224T/R316E), which displayed a maximal response of approximately 6-fold.

We then performed saturated mutagenesis on both residues together on L1 (FN) and L2 (GT) between TrpR and cpSFYFP. For the mutagenesis library of each linker, we changed the targeted codons (FN or GT) with NNSNNS in the primers and screened approximately 4,000 colonies for each linker to obtain improved mutants. We screened ~8,000 colonies and obtained 9 improved mutants of linker2 but no improved variant of linker1. According to the maximal fluorescence response, we chose the mutant GRIT0-L2-QA for *in vitro* characterization and denoted it GRIT (**Fig. 1a; Supplementary information, Fig. S1e**).

To generate a control sensor with no response to tryptophan (GRITOL), we conducted site-directed saturated mutagenesis on the key sites of the tryptophan binding pocket, including R54, V58 and R331 (R84 relative to TrpR)[6]. We identified a GRIT control sensor with R54G mutation (GRITOL), which displayed no fluorescence response to tryptophan (**Supplementary information, Fig. S1e**). The protein sequences of GRIT and GRITOL are listed in **Supplementary information, Note 1**.

Protein Expression and Purification

The vectors pRSETb-GRIT and pRSETb-GRITOL were transformed into JM109(DE3) competent *E. coli* cells. Single clone was grown in 100 ml LB media containing 100 μg/ml ampicillin at 37 °C until the absorbance at 600 nm of bacteria culture reached ~0.4. Protein expression was induced by addition of 1 mM IPTG at 20 °C for 24 hrs. Bacteria were spun down at 4,000 rpm for 30 min at 4 °C. Cell pellets were suspended in protein purification binding buffer (30 mM sodium phosphate, 500

mM sodium chloride, and 50 mM imidazole, 0.1 mg/ml lysozyme, 0.1% Triton X-100, 1 mM protease inhibitors cocktail, pH 7.4), and lysed with an ultrasonic cell disruptor (SCIENTZ Biotech). The supernatant was loaded on a NTA column (GE Healthcare) for affinity purification**.** The protein eluent was desalted in detection buffer (10 mM HEPES, 100 mM KCl, pH 7.3), quantified with a BCA kit (Mxbioscience LLC) and stored at -80 °C for measurements.

In Vitro Characterization of GRIT Sensor

For the excitation and emission spectra, 3 μM purified biosensors in the presence or absence with 0.5 mM tryptophan were placed in a quartz cuvette and monitored with a Cary Eclipse spectrofluorimeter (Varian).

For all microplate experiments, 50 μl amino acids and 50 μl protein were mixed in black 96-well flat-bottomed plate. The fluorescence intensities of 0.2 μM biosensors were measured immediately after addition of amino acids on a SynergyNeo Multi-Mode microplate reader equipped with an emission filter 528/20 and two excitation filters 420/10 and 485/20 (BioTek). The background intensity was subtracted, following which the values of excitation intensities or excitation ratios ($R_{485/420}$) were normalized. The apparent dissociation constant (Kd) is defined as the concentration of ligand, in which half the ligand binding sites on the protein are occupied in the system equilibrium. The detection range of the biosensor can be defined as the tryptophan concentrations that can induce a fluorescence response between 5% and 95% of the maximal response[7], thus the GRIT sensor could robustly detect a wide range of tryptophan concentration, spanning from 20 μM to 2.5 mM. Three independent measurements were averaged and fitted by SigmaPlot to obtain the apparent dissociate parameter. Correction is conducted by dividing the fluorescence ratio ($R_{485/420}$) of GRIT by that of GRITOL.

Plasmids Construction

For cytoplasmic expression, the coding sequences of GRIT and GRITOL with Kozak sequence (GCCACC) were cloned into the pcDNA3.1/hygro(+) vector with NheI and HindIII. To make lentiviral vectors, genes of cyto-GRIT and cyto-GRITOL were subcloned into pLVX-IRES-Puro backbone by NheI and PmeI. For construction of zebrafish transgenic plasmids, cyto-GRIT and cyto-GRITOL were amplified and ligated into the pTol2 vectors harboring *β-actin*, respectively.

Cell Culture, Transfections, and Establishment of Stable Cell Lines

HeLa cells and HEK293T cells were derived from the cell bank of Chinese Academy of Science and validated by mycoplasma contamination-free test. All cell lines were cultivated in high glucose DMEM (Hyclone) with 10% FBS (ExCell Bio) and 1% penicillin-streptomycin (10,000 U/ml) and maintained at 37 °C in a humidified atmosphere of 95% air and 5% CO_2.

For transient transfections, HeLa cells were plated on an 8-well glass bottomed dish (Cellvis) with ~70% confluency, followed by plasmid transfections with lipofectamine 3000 (Invitrogen) according to the manufacturer's protocol.

For lentivirus preparation, the lentiviral plasmids were co-transfected with two packaging vectors (pMD2.G and psPAX2) into HEK293T cells, which are pre-seeded on 60 mm tissue culture dishes with Hieff Trans (YEASEN). The lentivirus supernatants were harvested from cell culture at 48 and 72 hrs after transfection and applied to HeLa cells in 6-well tissue culture plates with 8 μg/ml polybrene. The stable cells were cultivated with 1 μg/ml puromycin for 1 week and sorted by MoFlo Astrios EQ Beckman Coulter) with laser line at 488 nm.

Fluorescence Measurement of Living Cells with Microplate Reader

The stable HeLa cell lines with GRIT sensors were trypsinized and counted with a hemocytometer. Cell pellets were resuspended and seeded into a black 96-well flat-bottom plate with 26,000 cells per well for 10-12 hrs cultivation. Cells were rinsed twice with freshly prepared HEPES buffer (150 mM NaCl, 1 mM $MgCl_2$, 1 mM $CaCl_2$, 10 mM D-Glucose, 10 mM HEPES, pH 7.4) to remove the DMEM media before detection. Then different amino acids were added in the detection buffer and their concentrations were listed as absolute concentrations. The detection conditions are similar to that in protein characterization. The signals of the HeLa cell samples were subtracted as background for correction of the fluorescence values of GRIT sensors.

Fluorescence Microscopy

HeLa cell expressing Cyto-GRIT and Cyto-GRITOL were plated on an 8-well glass bottomed dish (Cellvis) and the culture medium was changed to HEPES buffer containing tryptophan or histidine of different concentrations before experiment. For dual-excitation ratio imaging, images were acquired by an inverted confocal Olympus FV3000 automatic microscope with a UPlanSApo 40 × Sil objective (N.A. 1.25). The 405 nm and 488 nm laser were used to excite GRIT sensor and the 500-550 nm emission figures were captured by a photomultiplier tube (PMT) as 1024×1024 format, 12-bit depth.

All data were analyzed by ImageJ Fiji. The same cell in the 488 nm excitation image was divided by 405 nm excitation image and pseudocolored in the Hue-Saturation-Brightness (HSB) color bar. The values (255, 0, 0) and (255, 0, 255) represent the maximum and minimum excitation ratio, respectively.

In Vitro Transcription and mRNA Purification

The preparation of mRNA was performed as previously described[8]. Briefly, the cDNAs of GRIT, GRITOL or Tol2 were amplified by PCR with the promoter and terminator, followed by *in vitro* transcription with a T7 mMESSAGE mMACHINE kit (Invitrogen) according to the manufacturer's

manual. mRNA was purified by lithium chloride incubation for 2 hrs at -20 °C and diluted with the DEPC-treated nuclease-free water.

Transgenic Zebrafish Procedures and *In Vivo* Imaging

The transgenic zebrafish was prepared by the Tol2 transposase-based approach as reported previously[8]. To measure intracellular tryptophan levels in different tissues, 25 ng/μl plasmid DNA with the *β-actin* together with 25 ng/μL Tol2 transposase mRNA (1 nL) was injected into zebrafish embryos at one cell stage with an air-puffed MPPI-3 pressure injector (ASI). To detect tryptophan dynamics during inflammation, 1 nL mRNA mixture (170 ng GRIT or GRITOL sensor) was injected to generate fluorescent zebrafish larvae. For DNA and RNA microinjection, experiments were performed on either 6 day-post-fermentation (dpf) or 3 dpf larvae, respectively. The handling procedures were approved by Center for Excellence in Brain Science and Intelligence, Chinese Academy of Sciences.

For *in vivo* imaging, zebrafish larvae were paralyzed in sterilized egg water (1 mM HEPES, 5 mM NaCl, 0.17 mM KCl, 0.33 mM $CaCl_2$,0.33 mM $MgSO_4$, 1 mM pancuronium dibromide (Selleck), pH 7.4) and embedded dorsal side up into 1.5% low melting agarose on a glass-bottom dish under stereoscopic microscope. Zebrafish larvae were maintained on the Olympus FV3000 upright confocal microscope equipped with a XLUMPlanFL N 20X water-immersion objective (N.A. 1.0) for 20 min before imaging.

For GRIT sensor imaging, dual excitation laser at 405-nm and 488-nm and a 500-550 nm filter were used. In the time-lapse experiment, 5 mM tryptophan was added by pipette followed by the 3-hr z stack image capture with a view filed of 512 X 512 pixels. For still images in bath application experiments, the 30-slice image stacks with a view filed of 1024 X 1024 pixels were captured before and after addition of 5 mM tryptophan or 20 mM histidine. The acquired z stacks of both single timepoint and time lapse images were selected to encompass all ROIs (regions of interest) within the zebrafish larvae.

To observe zebrafish single cells at high resolution, images were taken at 1 μm axial step for 200 slices as 1024 X 1024 format to encompass the whole zebrafish expressing GRIT sensor. The excitation ratio images were processed and pseudocolored with imageJ Fiji as described above, followed by creation a 3D reconstruction of larval using Imaris (Bitplane, Belfast, United Kingdom).

Primary Zebrafish Cells Isolation

Primary zebrafish cell cultures were prepared as described previously[9]. Briefly, 25 ng/μL plasmid DNA (*β-actin*:Cyto-GRIT or *β-actin*:Cyto-GRITOL) and 170 ng/μL corresponding mRNA transcript mixed with 25 ng/μL Tol2 mRNA was microinjected to one-cell stage embryos. Zebrafish larvae at 2 dpf were washed twice by PBS and then anaesthetized in ice-cold PBS buffer with 0.6 mM tricaine for 1 min. The chopped zebrafish stumps were digested by 28 U/mL papain, 0.02 mg/mL DNase and 0.48 mg/mL L-cysteine in DMEM for 10 min. After digestion, the tissue slices were gently triturated

to release cells into pre-warmed fresh DMEM medium with 10% FBS. The cell suspensions were filtered through a cell strainer of 70 µm pore size (BD Bioscience) and spun down at 800 g for 5 min at room temperature. The cell pellets were diluted into L15 media with 10% FBS and 1% penicillin-streptomycin and seeded on the precoated plastic coverslips (50 µg/ml poly-L-Lysine, Sigma) in a 12-well plate. Primary cell cultures were grown in a water-jacketed incubator at 27 °C for 12 hrs.

Zebrafish Inflammatory Model

LPS induced inflammation in larval zebrafish was performed as described previously[10]. In brief, 2.5 dpf zebrafish larvae were anesthetized with 1 mM pancuronium dibromide in egg water, followed by the microinjection of 1 nL LPS (25 ng/nL) or 1 nL PBS vehicle into the yolk. The time-course z stack images were captured with the frequency of 1 frame/h as 512 X 512 format in 12-bit depth.

For zebrafish plasma tryptophan measurement, 1 nL purified 100 µM GRIT or GRITOL protein was microinjected into the common cardinal vein (CCV) of zebrafish larvae with desired treatments. Images were immediately captured after protein injection.

Fish line *Tg*(*mpeg1:GFP*) was used to track macrophage activation and transmigration[11]. The accumulation percentage was calculated by dividing the number of macrophages at the yolk to that of whole body.

To measure ROS production, the wild type Nacre zebrafish larvae were bathed with egg water containing 100 µM 2', 7'-dichlorodihydrofluorescein diacetate (DCFH-DA) for 1 h at room temperature in dark, followed by rinse 3 times before imaging with a PlanApo 2X objective (N.A. 0.8) on Olympus FV3000 confocal microscope.

Calibration of Intracellular and Extracellular Tryptophan Levels *Ex Vivo* and *In Vivo*

The fundamental assumption of quantitative measurement with the ratiometric biosensor GRIT is that the properties of the purified sensor could be used as a reference to measure tryptophan levels in cells and *in vivo*[12]. Thus, the quantitative measurement can be conducted by the calculation of normalized sensor fluorescence ratios in living cells and comparison with those of purified GRIT protein *in vitro,* as described previously[2].

For *in situ* live-cell calibration, the unbound and saturated states of the GRIT sensor in HeLa cells could be approximated by treatment with 5 mM histidine and 0.5 mM tryptophan in modified HEPES buffer (pH 7.4). The detection conditions were similar to those in live-cell fluorescence measurements. The cytosolic tryptophan levels were estimated by fitting with purified protein titration curves at pH 7.3. To account for the pH effect, the excitation ratios of cells expressing GRIT were corrected by those of cells expressing GRITOL measured in parallel and fitted with an *in vitro* protein titration curve at pH 7.3.

To calibrate tryptophan concentrations in zebrafish larvae, we isolated primary zebrafish cells and performed live-cell imaging by the addition of 0.5 mM tryptophan or 5 mM histidine. We ran z-stack imaging on an Olympus FV3000 confocal microscope with the orthographic view using the same settings in the zebrafish experiment. The z-stack images from 488 nm were projected and divided by the 405 nm z projection images. The excitation fluorescence ratios of cells *in vivo* were normalized and calibrated as described above.

Tryptophan Quantification with High Performance Liquid Chromatography

Intracellular tryptophan quantification was performed with LC/MS methods as reported[13]. In brief, HeLa cells were harvested, and the intracellular metabolites were extracted by addition of 80% pre-cold methanol. The supernatant was evaporated to dryness at 4°C using a vacuum concentrator, dissolved in 100 μL 90% acetonitrile to remove proteins and subjected to LC/MS quantification with a UHPLC system (1290 series; Agilent Technologies, USA) coupled to a quadruple time-of-flight mass spectrometer (TripleTOF 6600, AB SCIEX, USA). The results were analyzed with the Agilent MassHunter Qualitative Analysis Navigator (B.08) software.

The Procedure and Code for *In Vivo* Imaging Figure Analysis

Images were processed with the Fiji software and stitched with Grid/Collection stitching plugin. Background subtraction was conducted by either selection of a ROI or the rolling ball in any desired pixels. To remove the nonspecific skin fluorescence signals, all cells in zebrafish were circled in one ROI manually on the pseudocolored ratiometric image and the outside skins were cleared per slice. The ROIs of single cell particles could be obtained manually or semi-automatically. In the semi-automatic model, images were binarized in desired channel and split after filtering, followed by the ROI collection by customized particle parameters. The fluorescence intensities of all ROIs in each channel were measured and exported in a CSV file for further analysis.

Statistical Analysis

Data are presented either as a representative example of a single experiment repeated at least in triplicate or as the mean values of three or more experiments. All statistical tests and graphs were made with Sigmaplot, MATLAB or Python. The figures were assembled in Adobe illustrator 2020. Data are represented as the mean ± s.e.m.

In the zebrafish experiments, the normality test was performed before analysis of the equality of variances between two independent groups with Levene's test. If the value was less than 0.05, we calculated the significance level (P values) with Student's unpaired t test. Otherwise, we used Welch's t test. The specific sample size (n numbers) and P values of each figure in the zebrafish experiments are listed in the **Table S7**.

Code Availability

The code used for ratiometric fluorescence image analysis and a tutorial video for instructions are available at https://github.com/KeJiiii/FIQA/releases.

Data Availability

The data that supports the conclusions of this study are available from the corresponding author upon request. All constructs, including GRIT and GRITOL, are available on requests.

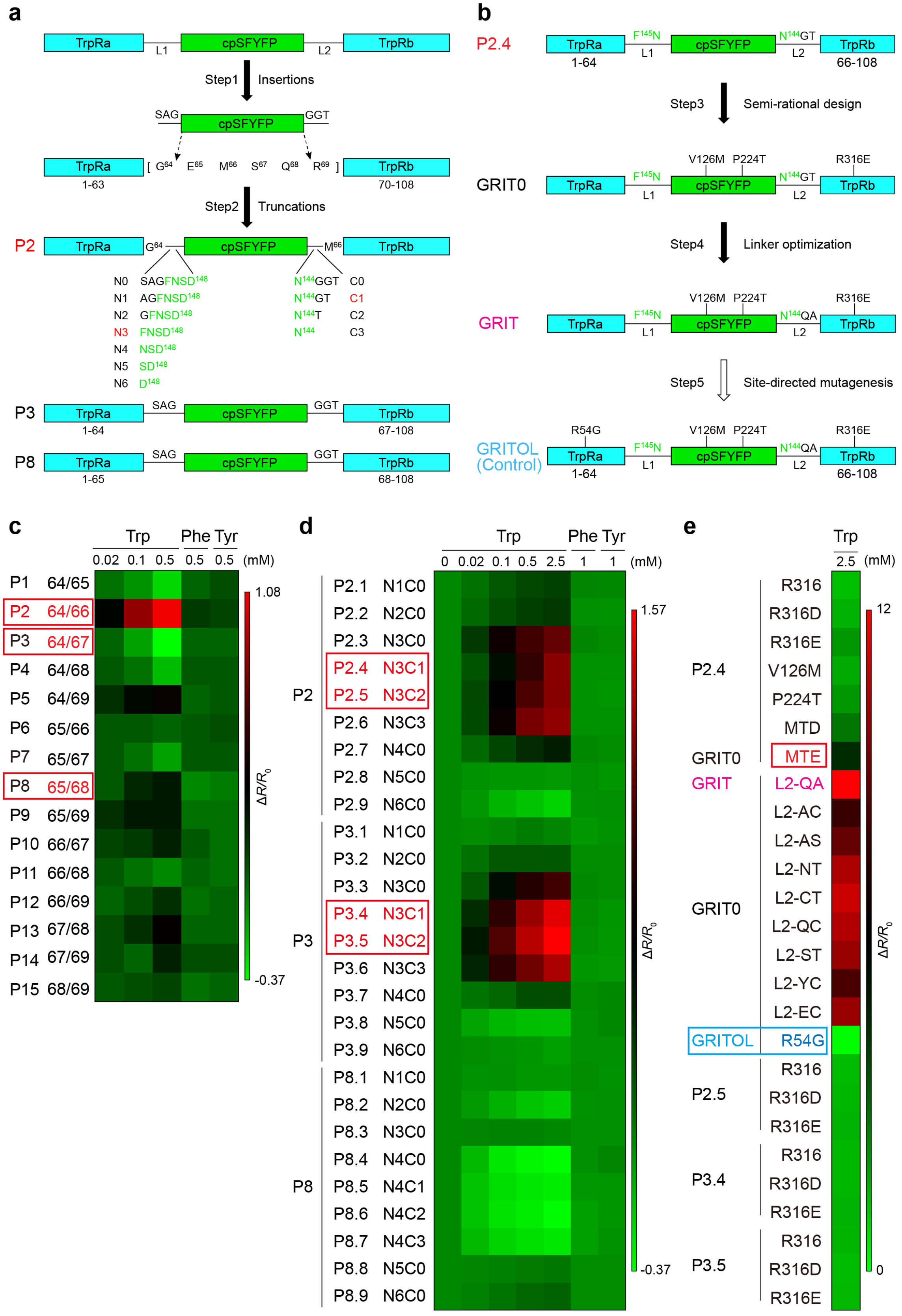

a
TrpRa
L1
cpSFYFP
L2
TrpRb
Step1
Insertions
SAG
GGT
1-63
70-108
Step2
Truncations
P2
N0 SAGFNSD148
N1 AGFNSD148
N2 GFNSD148
N3 FNSD148
N4 NSD148
N5 SD148
N6 D148
N144GGT C0
N144GT C1
N144T C2
N144 C3
P3
1-64
67-108
P8
1-65
68-108
b
P2.4
66-108
Step3
Semi-rational design
GRIT0
V126M P224T
R316E
Step4
Linker optimization
GRIT
Step5
Site-directed mutagenesis
GRITOL (Control)
R54G
c
Trp
Phe
Tyr
(mM)
P1 64/65
P2 64/66
P3 64/67
P4 64/68
P5 64/69
P6 65/66
P7 65/67
P8 65/68
P9 65/69
P10 66/67
P11 66/68
P12 66/69
P13 67/68
P14 67/69
P15 68/69
1.08
-0.37
ΔR/R0
d
P2.1 N1C0
P2.2 N2C0
P2.3 N3C0
P2.4 N3C1
P2.5 N3C2
P2.6 N3C3
P2.7 N4C0
P2.8 N5C0
P2.9 N6C0
P3.1 N1C0
P3.2 N2C0
P3.3 N3C0
P3.4 N3C1
P3.5 N3C2
P3.6 N3C3
P3.7 N4C0
P3.8 N5C0
P3.9 N6C0
P8.1 N1C0
P8.2 N2C0
P8.3 N3C0
P8.4 N4C0
P8.5 N4C1
P8.6 N4C2
P8.7 N4C3
P8.8 N5C0
P8.9 N6C0
1.57
-0.37
e
R316
R316D
R316E
V126M
P224T
MTD
MTE
L2-QA
L2-AC
L2-AS
L2-NT
L2-CT
L2-QC
L2-ST
L2-YC
L2-EC
R54G
P2.5
P3.4
P3.5
12
0

Supplementary information, Fig. S1: Generation of cpSFYFP-based tryptophan sensors. (a and b) Schematic for the development of GRIT sensor. **(c-e)** Fluorescence responses of three collections of tryptophan sensors to different doses of tryptophan (Trp), or Phe, Tyr; 15 insertions (c) from (a), 27 truncations based on P2, P3 and P8 (d) from (a), 26 representative mutants and control sensor (e) from (b). MTD and MTE mean the mutants harbored V126M, P224T, R316D or R316E, respectively. The biosensors for further optimization were highlighted in red and marked in a rectangle. GRIT sensor and GRITOL sensor were highlighted in magenta and blue, respectively.

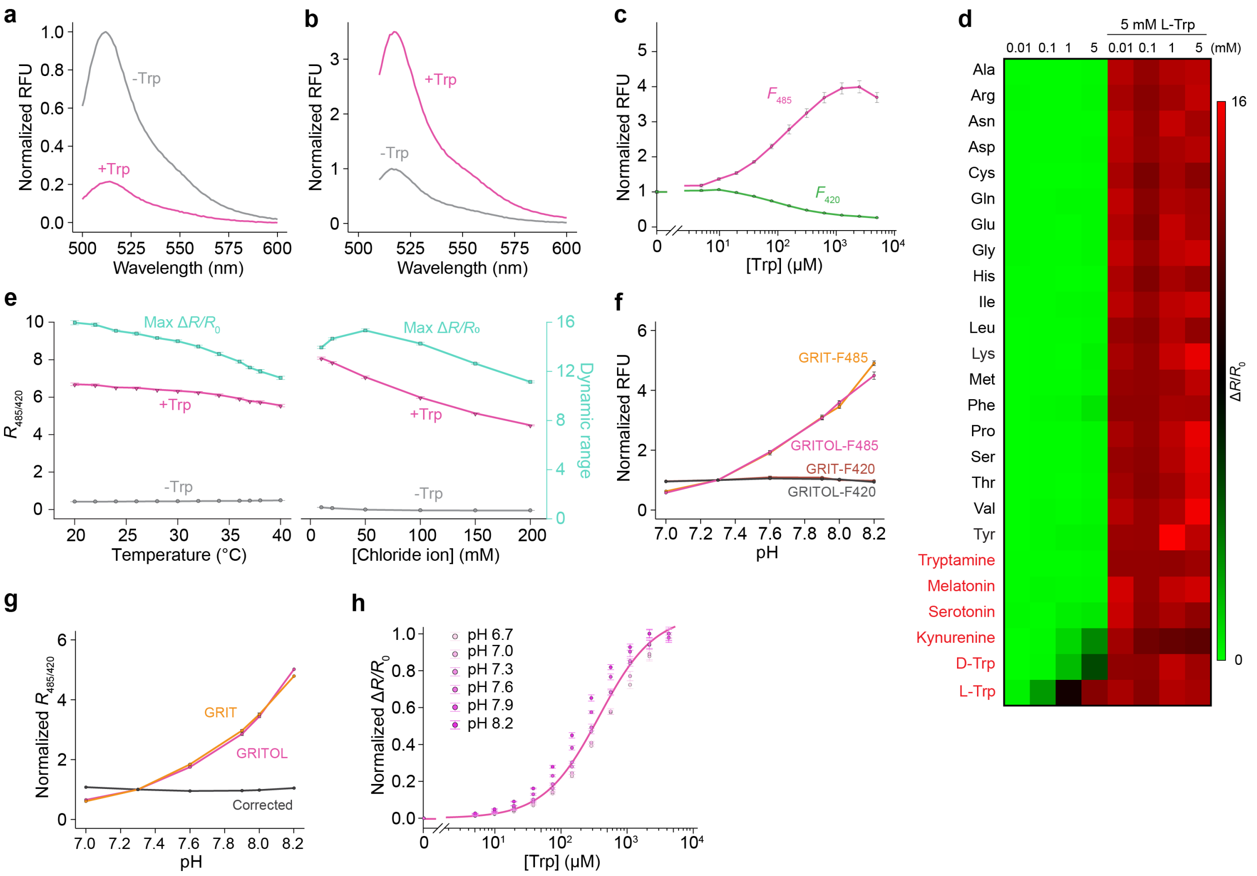

Supplementary information, Fig. S2: Characterization of purified GRIT *in vitro* and in living bacteria. (a and b) Emission spectra of GRIT in the absence (a) or presence of 1 mM Trp (b) normalized to the peak intensity in control conditions. Excitations were fixed at 420 nm (a) and 490 nm (b), respectively. **(c)** Emission intensities of GRIT excited at 420 nm or 485 nm in the presence of different concentrations of Trp. Data are normalized to the initial value. **(d)** The responses of GRIT to different doses of amino acids and tryptophan metabolites (tryptamine, melatonin, serotonin and kynurenine) or analog (D-Trp) in the absence or presence of 5 mM L-Trp. **(e)** Fluorescence responses of GRIT against 1 mM Trp at different temperatures or chloride solutions. **(f)** The effects of pH on the excitation intensities of GRIT and GRITOL at 420 nm or 485 nm. Data were normalized to the fluorescence at pH 7.3. **(g)** pH-dependence of the excitation ratios of GRIT and GRITOL. Data were normalized to the fluorescence ratio at pH 7.3. **(h)** Tryptophan dose-response curves of GRIT corrected by GRITOL at pH from 7.0 to 8.2. Data were normalized to 0 - 1. pH correction is performed by dividing the excitation ratio ($R_{485/420}$) of GRIT by that of GRITOL.

Error bars represent s.e.m., if not mentioned. For panel Fig. S2a-h, n = 3 - 5 experiments.

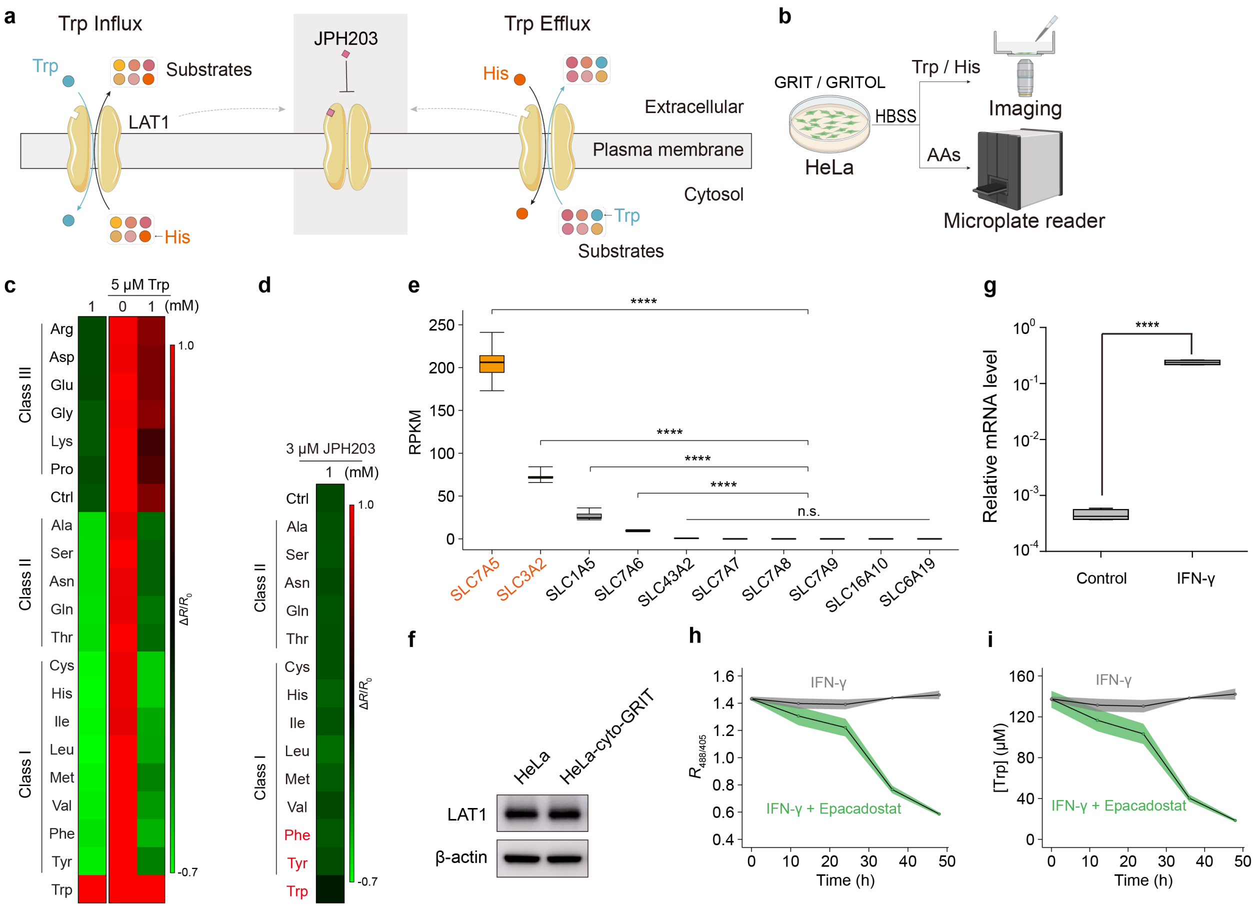

Supplementary information, Fig. S3: Detection of tryptophan dynamics in living HeLa cells. (a) The working model of the large neutral amino acids transporter (LAT1). LAT1 simultaneously uptake extracellular Trp into cytosol and export the other LAT1 substrates (left). In the presence of extracellular His, LAT1 could induce efflux of cytosolic Trp and other LAT1 substrates (right). Those processes are blocked by the antagonist JPH203 (middle). **(b)** Schematic showing fluorescence detection of the GRIT or GRITOL sensor in HeLa cells. (**c**) HeLa cell expressing cytosolic GRIT in response to indicated concentration of amino acids in the absence and presence of 5 µM tryptophan in HBSS buffer. **(d)** The effect of 3 µM SLC7A5 (LAT1) antagonist JPH203 on tryptophan transport in HeLa cells upon addition of indicated amino acids. Note that the concentration of all amino acids is 1 mM, except that the concentration of Phe, Tyr and Trp is 20 µM. **(e)** The expression level of all reported tryptophan transporters of HeLa cells. The SLC7A5 (LAT1) and its regulatory subunit SLC3A2 (4F2hc) were marked in orange (n = 9 - 18). Datasets are taken from previous reports[14]. **(f)** Immunoblot of the LAT1 expression level in HeLa cells and HeLa-cyto-GRIT cells. **(g)** The mRNA level of IDO1 in HeLa cells treated with or without 10 ng/mL IFN-γ stimulation for 48 hr. n = 6. **(h-i)** The fluorescence response of GRIT sensor (h) and cytosolic tryptophan level (i) in HeLa cells in response to IFN-γ stimulation with or without 1 µM IDO1 inhibitor Epacadostat.

Data shown as mean ± s.e.m. (n.s., non-significant; ****P < 0.0001). Student's unpaired *t* test for Fig. S3e, g. For panel Fig. S3b, c, d and f, n = 3-5 experiments.

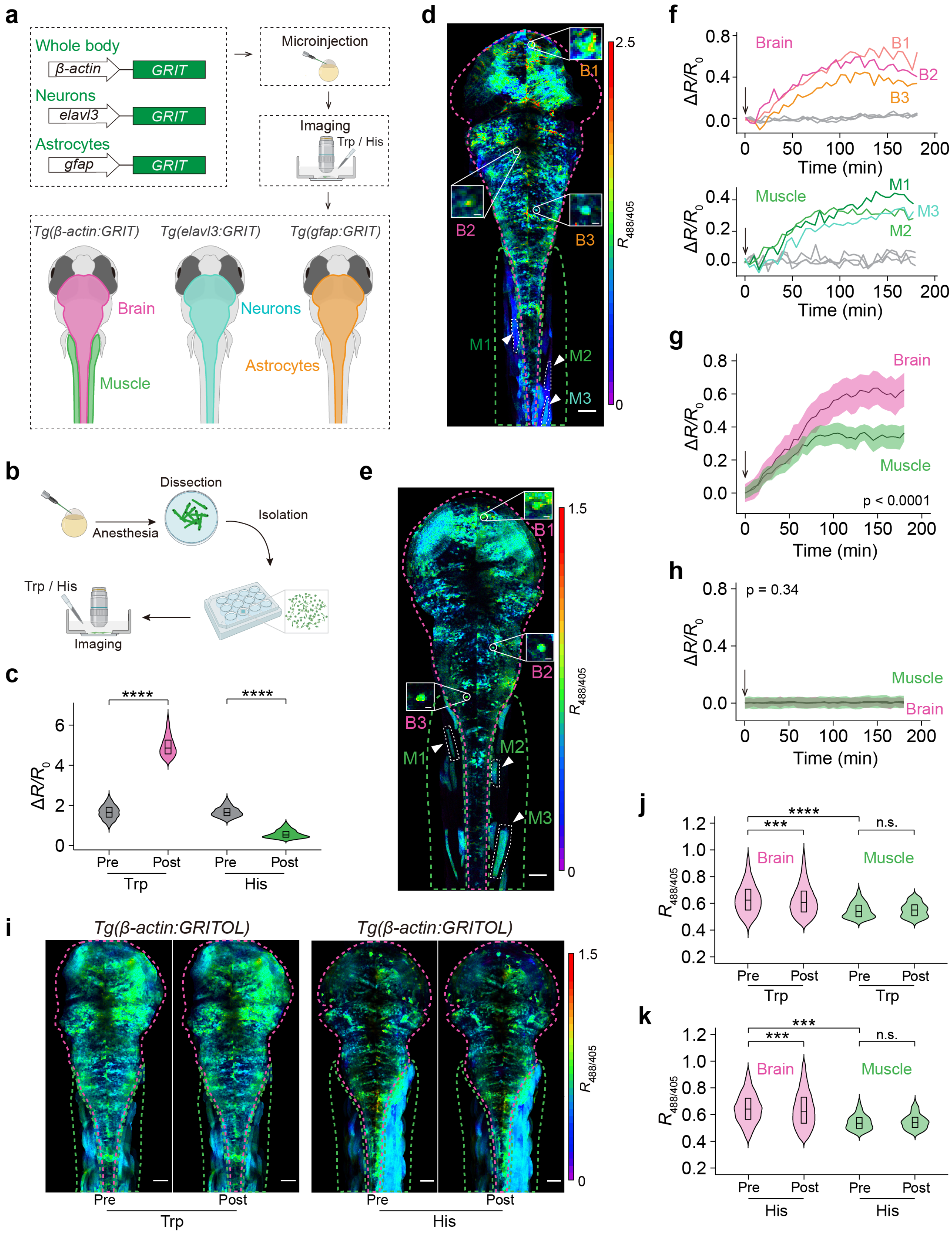
a
Whole body
β-actin
GRIT
Neurons
elavl3
GRIT
Astrocytes
gfap
GRIT
Microinjection
Imaging
Trp / His
Tg(β-actin:GRIT)
Tg(elavl3:GRIT)
Tg(gfap:GRIT)
Brain
Neurons
Muscle
Astrocytes
b
Dissection
Anesthesia
Isolation
Trp / His
Imaging
c
ΔR/R0
****
****
Pre
Post
Pre
Post
Trp
His
d
B1
B2
B3
M1
M2
M3
R488/405
2.5
0
e
B1
B2
B3
M1
M2
M3
R488/405
1.5
0
f
Brain
B1
B2
B3
ΔR/R0
Time (min)
Muscle
M1
M3
M2
g
Brain
Muscle
p < 0.0001
h
p = 0.34
Muscle
Brain
i
Tg(β-actin:GRITOL)
Tg(β-actin:GRITOL)
R488/405
1.5
0
Pre
Post
Trp
Pre
Post
His
j
R488/405
****
***
n.s.
Brain
Muscle
Pre
Post
Pre
Post
Trp
Trp
k
***
***
n.s.
Brain
Muscle
Pre
Post
Pre
Post
His
His

Supplementary information, Fig. S4: Visualization of tryptophan dynamics in primary zebrafish cells and zebrafish larvae. **(a)** Schematic of the experimental protocol for GRIT sensor imaging in zebrafish larvae. **(b)** Schematic figure showing the procedure of tryptophan calibration in primary zebrafish cell culture. **(c)** Fluorescence responses of GRIT sensor in primary zebrafish cells against 0.5 mM Trp or 5 mM histidine (His). n = 4 independent experiments. **(d-e)** Representative pseudocolor image (maximum projection along the z-axis) of GRIT-expressing zebrafish (d) or GRITOL-expressing zebrafish (e). White circles and arrowheads represent the selected single cells from the brain (B) and muscles (M), respectively. The circled single brain cells are zoomed in to show details. The dashed lines indicate the boundary of the brain (pink) and the muscles (green). **(f)** Representative traces (region of interest from d and e) of the normalized fluorescence changes in GRIT-expressing cells (d) or GRITOL-expressing cells (e) from the brain (upper) and muscles (bottom) in response to 5 mM tryptophan. Arrows indicate the addition of tryptophan. The slow kinetics of GRIT sensor *in vivo* may be related with the absorption of tryptophan from media and transport into tissue cells. **(g-h)** The mean fluorescence kinetics of GRIT (g) and GRITOL (h) in brain cells and muscle cells to exogenous tryptophan. **(i)** Fluorescent images of zebrafish expressing GRITOL sensor driven by *β-actin* promoter against 5 mM Trp or 20 mM His. **(j and k)** Group analysis of the excitation ratios of GRITOL cells in different regions treated with 5 mM Trp (j) or 20 mM His (k). Note that the small P-values in panel Fig. S4j and S4k do not necessarily imply the presence of large statistical difference, due to the large sample size[15]: the ROIs of brain, 10932-11894, muscles, 176-390.

Scale bars in panel Fig. S4d, 4e and 4i, 50 μm. For panel Fig. S4d-k, n = 4 fish. Data shown as mean ± s.e.m. (n.s., non-significant; ***P < 0.001, ****P < 0.0001). Two-way ANOVA for Fig. S4g, S4h and Student's unpaired *t* test for Fig. S4c, S4j and S4k. See also **Supplementary information, Table S2**. Detailed n number and P value are in **Table S7**.

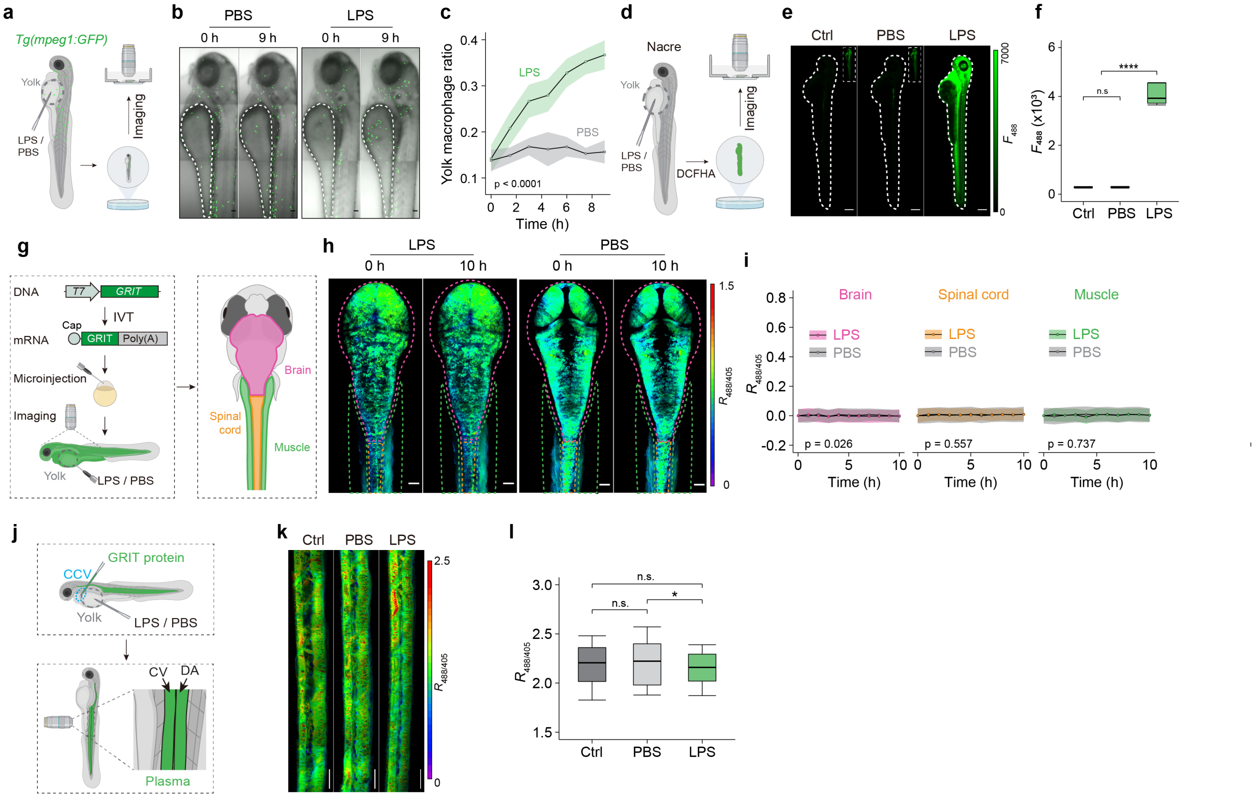

Supplementary information, Fig. S5: Visualization of tryptophan dynamics under LPS-induced inflammation in zebrafish larvae. **(a)** Schematic for LPS-induced inflammation in *Tg*(*mpeg1:GFP*) zebrafish larvae. **(b)** Representative merged images of *Tg*(*mpeg1:GFP*) zebrafish larvae before (0 h) and after (9 h) injection with LPS (n = 16) or PBS vehicle (n = 7). The yolks were marked in dotted circles. **(c)** The proportion of accumulated macrophages in the yolk to body macrophages of zebrafish larvae after LPS or PBS injection. The percentage is calculated by dividing the macrophage number of the yolk in the same fish at the same timepoint by that of the body. **(d)** Schematic of ROS measurement by DCFHA in zebrafish larvae under LPS-induced inflammation. **(e and f)** Confocal imaging (e) and mean fluorescence intensities (f) of zebrafish larvae staining with DCFHA after treated with LPS (n = 12) or PBS (n = 8). Noted that the left and medium fish were too dim and zoomed-in with the calibration bar 0 - 600. Scale bar, 200 µm. **(g)** Schematic of LPS-induced inflammation in zebrafish larvae. **(h)** Fluorescent images (maximum projection along z-axis) of zebrafish expressing GRITOL before (0 h) and after (10 h) indicated treatments. **(i)** The normalized mean fluorescence responses of GRITOL sensor in the brain, spinal cord and muscles after injection with LPS or PBS vehicle. **(j)** Schematic depicting the *in vivo* detection of plasma tryptophan levels with purified GRIT protein in the caudal vein (CV) and dorsal aorta (DA). **(k and l)** Representative fluorescence images (k, maximum projection along z-axis) and the

averaged excitation ratios (l) of injected GRITOL sensor into plasma of zebrafish with indicated treatments.

Scale bars in panel Fig. S5b, 5h and 5k, 50 μm. For panel Fig. S5i and 5l, n = 4 - 6 fish. Data shown as mean ± s.e.m. (n.s., non-significant; *$P < 0.05$, ****$P < 0.0001$). Two-way ANOVA for Fig. S5c, S5i and Student's unpaired *t* test for Fig. S5f and 5l. See also **Supplementary information, Table S4**. Detailed n number and P value are in **Table S7**.

Supplementary information, Table S1: The photophysical properties of GRIT sensor in the absence or presence of Trp. Photophysical properties of GRIT sensor with or without Trp were measured at room temperature. Extinction coefficients (ε, $mM^{-1} \cdot cm^{-1}$) were calculated from the absorbance at the excitation peaks (420 nm and 500 nm). The quantum yields (QY) of GRIT sensor were calculated using the reference EGFP at pH 7.3 (QY 0.60)[16]. Brightness is defined as the product of extinction coefficient and quantum yield. Data are mean ± s.e.m. from 3 independent experiments. The response kinetics were measured by an SX20 Stopped-Flow accessory (Applied Photophysics Ltd) as previously reported[17]. The apparent association (k_{on}) and dissociation (k_{off}) rate constants were 0.33 $\mu M^{-1}s^{-1}$ and 246 s^{-1}, respectively.

[Trp] (mM)	λ (nm)	ε ($mM^{-1} \cdot cm^{-1}$)	QY	Brightness
0	420	33.04 ± 0.21	0.18	6.1
0	500	25.14 ± 0.36	0.16	4.0
4	420	7.63 ± 0.22	0.18	1.3
4	500	26.20 ± 0.19	0.55	14.4

Supplementary information, Table S2: The maximal fluorescence responses of GRIT sensor in solutions, bacteria, mammalian cells and zebrafish. [a]Live HeLa cells were incubated with Trp or histidine (His); [b]GRIT sensor in intact zebrafish tissue in response to Trp or His; [c]Fluorescence responses of FLIPW-CTCY, taken from previous study[18].

Conditions	$\Delta R/R_0$		Fold increase
	GRIT	FLIPW-CTCY	
Purified protein (pH 7.3)	12.94 ± 0.24	~0.3[c]	~43
HeLa cytosol[a]	9.55 ± 0.43	0.1 - 0.3[c]	~32
Primary zebrafish cell	7.50 ± 0.19	ND	
Zebrafish tissue[b]	1.69 ± 0.09	ND	

Supplementary information, Table S3: Quantifications of tryptophan level in bacteria, mammalian cells, and zebrafish. [a]Cytosolic and mitochondrial tryptophan levels in HeLa cells or primary zebrafish cells were measured in HEPES buffer; [b]Reported tryptophan level in cultured COS-7 cells, taken from[18]; [c]Reported tryptophan level in cultured CHO cells, taken from[19]; [d]Reported tryptophan level in cultured HeLa cells, taken from[20]; [e]Tryptophan levels were measured by HPLC/MS in this study; [f]Saturated and [g]free state of GRIT sensor in HeLa cells were achieved by incubation with Trp or His, respectively.

Conditions	[Trp] (μM)				Uptake rate (μM/min)
	Control	Reference	Trp addition	His addition	
HeLa cytosol	157.2 ± 17.4[a]	340[b]<br>270 - 600[c]<br>330 – 473[d]<br>207.3 ± 8.2[e]	~100%[f]	~0%[g]	52.6 ± 13
Primary zebrafish cell cytosol	165.9 ± 8.3[a]		~100%	~0%	ND
Zebrafish brain	206.1 ± 21.8		494.4 ± 64.1	137.9 ± 13.8	ND
Zebrafish muscles	111.0 ± 6.5		211.7 ± 19.7	77.8 ± 7.9	ND
Zebrafish plasma	68.1 ± 3.8		ND	ND	ND

Supplementary information, Table S4: The effect of 19 amino acids on the tryptophan dynamics in cytosol and mitochondria of HeLa cells. The rate is defined by tryptophan concentration decrease per minute (μM/min), which is the sum of the efflux rate, endogenous consumption rate and tryptophan transportation rate into other subcellular organelles.

Conditions		Rate (μM/min)
	Control	2.12 ± 0.33
Class I	Cys	36.90 ± 2.41
	His	37.73 ± 1.58
	Ile	36.84 ± 2.47
	Leu	37.68 ± 1.63
	Met	37.05 ± 2.26
	Phe	35.05 ± 2.54
	Tyr	36.20 ± 2.80
	Val	36.09 ± 1.88
Class II	Ala	6.79 ± 0.42
	Ser	6.85 ± 0.37
	Asn	9.18 ± 0.54
	Gln	14.03 ± 0.56
	Thr	13.75 ± 0.76
Class III	Arg	1.83 ± 0.93
	Asp	1.81 ± 0.83
	Glu	1.76 ± 0.78
	Gly	2.34 ± 0.78
	Lys	2.33 ± 0.50
	Pro	1.91 ± 0.67

Supplementary information, Table S5: Estimated tryptophan level in zebrafish tissue and plasma.

	[Trp] (μM)		
Conditions	Zebrafish brain	Zebrafish muscles	Zebrafish spine cord
LPS-Pre	267.3 ± 22.1	114.5 ± 4.9	167.5 ± 8.7
LPS-Post	430.2 ± 38.5	144.3 ± 8.4	190.1 ± 9.3
PBS-Pre	256.0 ± 12.8	119.6 ± 4	174.8 ± 8
PBS-Post	248.1 ± 11.6	119.0 ± 4.3	174.2 ± 8.2
	Zebrafish plasma		
Ctrl	68.1 ± 3.8		
Inflammation	31.6 ± 3		
PBS	63.7 ± 3.5		

Supplementary information, Table S6: A comprehensive comparison between different tryptophan measurement methods, including HPLC/MS, electrochemical, FLIPW and GRIT.

		**HPLC/MS**	**Electrochemical**	**FLIPW**	**GRIT**
Detection range		0.01-100 μM	0.01 - 1 μM[21]<br>0.2 - 25 μM[22]<br>0.1 - 1.0 mM[23]	15 μM - 1 mM[18]	20 μM – 2.5 mM (this study)
Resolution	Spatial	No	No	Cell	Cell and plasma
	Temporal	Time point	Time point	Kinetics	Kinetics
Experimental process	Noninvasive	No	No	Yes	Yes
	Pre-preparation	Homogenate	Electrode	No	No
	Time-consumption	Long	Medium	Short	Short
	Detection speed	Slow	Medium	Fast	Fast
	Operation difficulty	Hard	Hard	Easy	Easy
Application examples	Solutions	Culture medium and serum[24-27]	Culture medium and serum[28]	Buffer	Buffer and plasma
	Cell	Cell and tissue Homogenate[29, 30]	Cell and tissue Homogenate [31]	Cultured COS[18]	Cultured HeLa, cultured primary zebrafish cells
	In vivo	N.A.	Mice[32]	N.A.	Zebrafish
Others	Throughput	Low	Low	High	High

Supplementary information, Table S7: Statistical analysis of distribution and uptake of tryptophan in zebrafish larvae

Panel	Category	Treatment	No. of dissections	Total No. of fish	Group	No. of cells
Fig. S4c	primary zebrafish cells from *Tg(β-actin: GRIT)*	Trp	4	600-800	Pre	2714
		Trp			Post	1841
		His			Pre	2187
		His			Post	3075
	primary zebrafish cells from *Tg(β-actin: GRITOL)*	Trp	4	600-800	Pre	936
		Trp			Post	1020
		His			Pre	1037
		His			Post	805

Panel	Category	Treatment	Group statistics	P value	Method
Fig. S4c	primary zebrafish cells from *Tg(β-actin: GRIT)*	Trp	Pre-Post	0	Student's unpaired t-test
		His	Pre-Post	0	
	primary zebrafish cells from *Tg(β-actin: GRITOL)*	Trp	Pre-Post	0.326571694	Student's unpaired t-test
		His	Pre-Post	0.005478154	

Panel	Category	Treatment	No. of fish	No. of slices	Timepoints	Group	No. of particles
Fig. S4g	*Tg(β-actin: GRIT)*	Trp	4	3	37	Brain	1230-1763
						Muscles	156-298
Fig. 1j	*Tg(β-actin: GRIT)*	Trp	4	20	1	Brain (Pre)	11894
						Muscles (Pre)	268
					1	Brain (Post)	11237
						Muscles (Post)	176
Fig. 1k		His	4		1	Brain (Pre)	11506
						Muscles (Pre)	377
					1	Brain (Post)	10932
						Muscles (Post)	390
Fig. S4h	*Tg(β-actin: GRITOL)*	Trp	4	3	37	Brain	1239-1845
						Muscles	285-316
Fig. S4j	*Tg(β-actin: GRITOL)*	Trp	3	20	1	Brain (Pre)	6167
						Muscles (Pre)	214
						Brain (Post)	6977
						Muscles (Post)	212
Fig. S4k		His	3			Brain (Pre)	8718
						Muscles (Pre)	212
						Brain (Post)	8309
						Muscles (Post)	230

Panel	Category	Treatment	Group statistics	P value	Method
Fig. S4g	*Tg(β-actin: GRIT)*	Trp	Brain-Muscles	5.872351e-318	Two-way ANOVA
Fig. 1j	*Tg(β-actin: GRIT)*	Trp	Brain: Pre-Post	0	Student's unpaired t-test
			Muscles: Pre-Post	1.20E-53	
			Pre: Brain-Muscles	7.80E-213	
Fig. 1k		His	Brain: Pre-Post	0	
			Muscles: Pre-Post	6.45E-87	
			Pre: Brain-Muscles	0	
Fig. S4h	*Tg(β-actin: GRITOL)*	Trp	Brain-Muscles	0.338727	Two-way ANOVA
Fig. S4j	*Tg(β-actin: GRITOL)*	Trp	Brain: Pre-Post	7.35E-07	Student's unpaired t-test
			Muscles: Pre-Post	0.501755096	
			Pre: Brain-Muscles	4.03E-60	
Fig. S4k		His	Brain: Pre-Post	0.001345148	
			Muscles: Pre-Post	0.353691206	
			Pre: Brain-Muscles	1.20E-182	

Statistical analysis of the tryptophan level, ROS level and macrophage activation ratio in zebrafish larvae under LPS-induced inflammation

Panel	Category	Indicator	Parameter	Treatment	No. of fish	No. of slices	Timepoints
Fig. S5c	*Tg(mpeg1:eGFP)*	eGFP	macrophage activation ratio	PBS	7	20	7
				LPS	16		
Fig. S5f	*nacre*	DCFH-DA	ROS	Ctrl	8	2	1
				PBS	8		
				LPS	12		

Panel	Category	Indicator	Parameter	Group statistics	P value	Method
Fig. S5c	*Tg(mpeg1:eGFP)*	eGFP	macrophage activation ratio	PBS-LPS	1.24E-08	Two-way ANOVA
Fig. S5f	*nacre*	DCFH-DA	ROS	Ctrl-PBS	0.731738444	Student's unpaired t-test
				Ctrl-LPS	8.63E-16	
				PBS-LPS	7.97E-16	

Panel	Category	Treatment	No. of fish	No. of slices	Timepoints	Group	No. of particles
Fig. 1m	GRIT mRNA	PBS	6	20	11	Brain	1065-1265
						Spinal cord	427-523
						Muscles	310-384
		LPS	8			Brain	1061-1461
						Spinal cord	403-542
						Muscles	335-441
Fig. 1o	GRIT Protein	Ctrl	4		1	Plasma	91
		PBS	3				80
		LPS	5				121
Fig. S5i	GRITOL mRNA	PBS	6	20	11	Brain	1483-1769
						Spinal cord	398-479
						Muscless	238-364
		LPS	6			Brain	1251-1461
						Spinal cord	449-542
						Muscless	352-441
Fig. S5l	GRITOL Protein	Ctrl	4		1	Plasma	116
		PBS	4				127
		LPS	4				124

Panel	Category	Group	Group statistics	P value	Method
Fig. 1m	GRIT mRNA	Brain	PBS-LPS	0	Two-way ANOVA
		Spinal cord	PBS-LPS	2.74E-15	
		Muscles	PBS-LPS	1.42E-42	
Fig. 1o	GRIT Protein	Plasma	Ctrl-PBS	0.358103777	Student's unpaired t-test
			Ctrl-LPS	6.01E-44	
			PBS-LPS	6.47E-40	
Fig. S5i	GRITOL mRNA	Brain	PBS-LPS	0.025583	Two-way ANOVA
		Spinal cord	PBS-LPS	0.557175	
		Muscless	PBS-LPS	0.737212	
Fig. S5l	GRITOL Protein	Plasma	Ctrl-PBS	0.353290112	Student's unpaired t-test
			Ctrl-LPS	0.428023774	
			PBS-LPS	0.048552303	

Supplementary information, Note 1: The sequence information.

GRIT sensor amino acid sequence:

TrpR(1-64)-cpSFYFP-L2-TrpR(67-108)

MAQQSPYSAAMAEQRHQEWLRFVDLLKNAYQNDLHLPLLNLMLTPDEREALGTRVRIVEELLR
GFNSDNVYIMADKQKNGIKANFKIRHNVEDGSVQLADHYQQNTPIGDGPVLLPDNHYLSFQSML
SKDPNEKRDHMVLLEFVTAAGITLGMDELYNVDGGSGGTGSKGEELFTGVVPILVELDGDVNG
HKFSVRGEGEGDATNGKLTLKLICTTGKLPVPWTTLVTTLGYGLKCFARYPDHMKQHDFFKSA
MPEGYVQERTIFFKDDGTYKTRAEVKFEGDTLVNRIELKGIGFKEDGNILGHKLEYNQAMSQEE
LKNELGAGIATITRGSNSLKAAPVELRQWLEEVLLKSD

GRITOL sensor amino acid sequence:

TrpR(1-64)-cpSFYFP-L2-TrpR(67-108)-R54G

MAQQSPYSAAMAEQRHQEWLRFVDLLKNAYQNDLHLPLLNLMLTPDEREALGTGVRIVEELLR
GFNSDNVYIMADKQKNGIKANFKIRHNVEDGSVQLADHYQQNTPIGDGPVLLPDNHYLSFQSML
SKDPNEKRDHMVLLEFVTAAGITLGMDELYNVDGGSGGTGSKGEELFTGVVPILVELDGDVNG
HKFSVRGEGEGDATNGKLTLKLICTTGKLPVPWTTLVTTLGYGLKCFARYPDHMKQHDFFKSA
MPEGYVQERTIFFKDDGTYKTRAEVKFEGDTLVNRIELKGIGFKEDGNILGHKLEYNQAMSQEE
LKNELGAGIATITRGSNSLKAAPVELRQWLEEVLLKSD

Supplementary References

1 Pedelacq JD, Cabantous S, Tran T, Terwilliger TC, Waldo GS. Engineering and characterization of a superfolder green fluorescent protein. *Nat Biotechnol* 2006; **24**:79-88.
2 Tao RK, Zhao YZ, Chu HY *et al.* Genetically encoded fluorescent sensors reveal dynamic regulation of NADPH metabolism. *Nat Methods* 2017; **14**:720-728.
3 Tao R, Shi M, Zou Y *et al.* Multicoloured fluorescent indicators for live-cell and in vivo imaging of inorganic mercury dynamics. *Free Radic Biol Med* 2018; **121**:26-37.
4 Belousov VV, Fradkov AF, Lukyanov KA *et al.* Genetically encoded fluorescent indicator for intracellular hydrogen peroxide. *Nat Methods* 2006; **3**:281-286.
5 Zhao Y, Araki S, Wu J *et al.* An expanded palette of genetically encoded Ca(2)(+) indicators. *Science* 2011; **333**:1888-1891.
6 He JJ, Matthews KS. Effect of amino acid alterations in the tryptophan-binding site of the trp repressor. *J Biol Chem* 1990; **265**:731-737.
7 Armbruster DA, Pry T. Limit of blank, limit of detection and limit of quantitation. *Clin Biochem Rev* 2008; **29 Suppl 1**:S49-52.
8 Kawakami K. Transposon tools and methods in zebrafish. *Dev Dyn* 2005; **234**:244-254.
9 Zhang H, Wang H, Shen X *et al.* The landscape of regulatory genes in brain-wide neuronal phenotypes of a vertebrate brain. *Elife* 2021; **10**.
10 Hsu AY, Gurol T, Sobreira TJP *et al.* Development and Characterization of an Endotoxemia Model in Zebra Fish. *Front Immunol* 2018; **9**:607.
11 Li D, Xue W, Li M *et al.* VCAM-1(+) macrophages guide the homing of HSPCs to a vascular niche. *Nature* 2018; **564**:119-124.
12 Park JG, Palmer AE. Quantitative measurement of Ca2+ and Zn2+ in mammalian cells using genetically encoded fluorescent biosensors. *Methods Mol Biol* 2014; **1071**:29-47.
13 Ser Z, Liu X, Tang NN, Locasale JW. Extraction parameters for metabolomics from cultured cells. *Anal Biochem* 2015; **475**:22-28.
14 Liu Y, Mi Y, Mueller T *et al.* Multi-omic measurements of heterogeneity in HeLa cells across laboratories. *Nat Biotechnol* 2019; **37**:314-322.
15 Nakagawa S, Cuthill IC. Effect size, confidence interval and statistical significance: a practical guide for biologists. *Biol Rev Camb Philos Soc* 2007; **82**:591-605.
16 Patterson GH, Knobel SM, Sharif WD, Kain SR, Piston DW. Use of the green fluorescent protein and its mutants in quantitative fluorescence microscopy. *Biophys J* 1997; **73**:2782-2790.
17 Wang L, Wu C, Peng W *et al.* A high-performance genetically encoded fluorescent indicator for in vivo cAMP imaging. *Nat Commun* 2022; **13**:5363.
18 Kaper T, Looger LL, Takanaga H, Platten M, Steinman L, Frommer WB. Nanosensor detection of an immunoregulatory tryptophan influx/kynurenine efflux cycle. *PLoS Biol* 2007; **5**:e257.
19 Heidemann R, Lutkemeyer D, Buntemeyer H, Lehmann J. Effects of dissolved oxygen levels and the role of extra- and intracellular amino acid concentrations upon the metabolism of mammalian cell lines during batch and continuous cultures. *Cytotechnology* 1998; **26**:185-197.
20 Chen WW, Freinkman E, Wang T, Birsoy K, Sabatini DM. Absolute Quantification of Matrix Metabolites Reveals the Dynamics of Mitochondrial Metabolism. *Cell* 2016; **166**:1324-1337 e1311.

21 Alam I, Lertanantawong B, Sutthibutpong T, Punnakitikashem P, Asanithi P. Molecularly Imprinted Polymer-Amyloid Fibril-Based Electrochemical Biosensor for Ultrasensitive Detection of Tryptophan. *Biosensors* 2022; **12**:291.
22 Zhang JW, Zhang X. Electrode material fabricated by loading cerium oxide nanoparticles on reduced graphene oxide and its application in electrochemical sensor for tryptophan. *Journal of Alloys and Compounds* 2020; **842**:155934.
23 Zor E, Hatay Patir I, Bingol H, Ersoz M. An electrochemical biosensor based on human serum albumin/graphene oxide/3-aminopropyltriethoxysilane modified ITO electrode for the enantioselective discrimination of d- and l-tryptophan. *Biosensors and Bioelectronics* 2013; **42**:321-325.
24 Ohashi H, Iizuka H, Yoshihara S *et al.* Determination of l-tryptophan and l-kynurenine in human serum by using LC-MS after derivatization with (R)-DBD-PyNCS. *International Journal of Tryptophan Research* 2013; **6**:IJTR. S11459.
25 Lionetto L, Ulivieri M, Capi M *et al.* Increased kynurenine-to-tryptophan ratio in the serum of patients infected with SARS-CoV2: An observational cohort study. *Biochimica et Biophysica Acta (BBA)-Molecular Basis of Disease* 2021; **1867**:166042.
26 Mathiesen L, Bay-Richter C, Wegener G, Liebenberg N, Knudsen LE. Maternal stress and placental function; ex vivo placental perfusion studying cortisol, cortisone, tryptophan and serotonin. *Plos one* 2020; **15**:e0233979.
27 Albu C, Radu LE, Radu G-L. Assessment of melatonin and its precursors content by a HPLC-MS/MS method from different Romanian wines. *ACS omega* 2020; **5**:27254-27260.
28 Nasimi H, Madsen JS, Zedan AH, Malmendal A, Osther PJS, Alatraktchi FAa. Electrochemical sensors for screening of tyrosine and tryptophan as biomarkers for diseases: A narrative review. *Microchemical Journal* 2023:108737.
29 Wang Y, Zhu W, Qiu J *et al.* Monitoring tryptophan metabolism after exposure to hexaconazole and the enantioselective metabolism of hexaconazole in rat hepatocytes in vitro. *Journal of Hazardous Materials* 2015; **295**:9-16.
30 Klupczynska A, Sulej-Suchomska AM, Piotrowska-Kempisty H, Wierzchowski M, Jodynis-Liebert J, Kokot ZJ. Development and validation of HPLC-MS/MS procedure for determination of 3, 4, 4′ , 5-tetra-methoxystilbene (DMU-212) and its metabolites in ovarian cancer cells and culture medium. *Journal of Chromatography B* 2017; **1060**:30-35.
31 Sadok I, Tyszczuk-Rotko K, Mroczka R, Staniszewska M. Simultaneous voltammetric analysis of tryptophan and kynurenine in culture medium from human cancer cells. *Talanta* 2020; **209**:120574.
32 Ling W, Liew G, Li Y *et al.* Materials and techniques for implantable nutrient sensing using flexible sensors integrated with metal–organic frameworks. *Advanced Materials* 2018; **30**:1800917.
